# Essentials of Physics

**Published:** 1892-09

**Authors:** 


ESSENTIALS OF PHYSICS.—Arranged in the form of questions and answers. Prepared especially for students of medicine, by Fred. J. Brockway, M. D., Assistant Demonstrator of Anatomy at the College of Physicians and Surgeons, New York. Published by W. B. Saunders, Philadelphia, Pa.

IS A VERY modest and highly instructive treatise on physics, and is, as its name implies, most admirably adapted to medical students. Though this little book does not aspire to the dignity of an exhaustive dissertation on physics, it will be found full of merit and worth, though modest. The price of $1.00 is cheap, and any one, whether medical student or not, will do well to read it. H. H.

